# Electroconductivity, a regenerative engineering approach to reverse rotator cuff muscle degeneration

**DOI:** 10.1093/rb/rbad099

**Published:** 2023-11-11

**Authors:** Nikoo Saveh-Shemshaki, Mohammed A Barajaa, Takayoshi Otsuka, Elnaz S Mirdamadi, Lakshmi S Nair, Cato T Laurencin

**Affiliations:** The Cato T. Laurencin Institute for Regenerative Engineering, University of Connecticut, Farmington, CT 06030, USA; Department of Biomedical Engineering, University of Connecticut, Storrs, CT 06269, USA; Department of Biomedical Engineering, Imam Abdulrahman Bin Faisal University, Dammam 31451, Saudi Arabia; The Cato T. Laurencin Institute for Regenerative Engineering, University of Connecticut, Farmington, CT 06030, USA; The Cato T. Laurencin Institute for Regenerative Engineering, University of Connecticut, Farmington, CT 06030, USA; Department of Biomedical Engineering, University of Connecticut, Storrs, CT 06269, USA; The Cato T. Laurencin Institute for Regenerative Engineering, University of Connecticut, Farmington, CT 06030, USA; Department of Biomedical Engineering, University of Connecticut, Storrs, CT 06269, USA; Department of Orthopedic Surgery, University of Connecticut Health Center, Farmington, CT 06030, USA; Department of Materials Science and Engineering, University of Connecticut, Storrs, CT 06269, USA; The Cato T. Laurencin Institute for Regenerative Engineering, University of Connecticut, Farmington, CT 06030, USA; Department of Biomedical Engineering, University of Connecticut, Storrs, CT 06269, USA; Department of Orthopedic Surgery, University of Connecticut Health Center, Farmington, CT 06030, USA; Department of Materials Science and Engineering, University of Connecticut, Storrs, CT 06269, USA; Department of Chemical & Biomolecular Engineering, University of Connecticut, Storrs, CT 06269, USA

**Keywords:** Skeletal muscle, Rotator Cuff, Electroconductivity, Regenerative engineering, scaffolds

## Abstract

Muscle degeneration is one the main factors that lead to the high rate of retear after a successful repair of rotator cuff (RC) tears. The current surgical practices have failed to treat patients with chronic massive rotator cuff tears (RCTs). Therefore, regenerative engineering approaches are being studied to address the challenges. Recent studies showed the promising outcomes of electroactive materials (EAMs) on the regeneration of electrically excitable tissues such as skeletal muscle. Here, we review the most important biological mechanism of RC muscle degeneration. Further, the review covers the recent studies on EAMs for muscle regeneration including RC muscle. Finally, we will discuss the future direction toward the application of EAMs for the augmentation of RCTs.

## Graphical abstract

**Figure:**
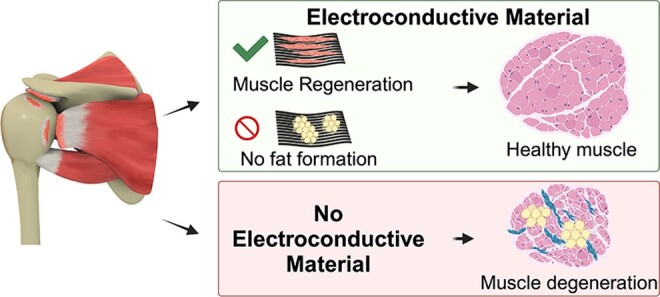

## Introduction

**Figure 1.**
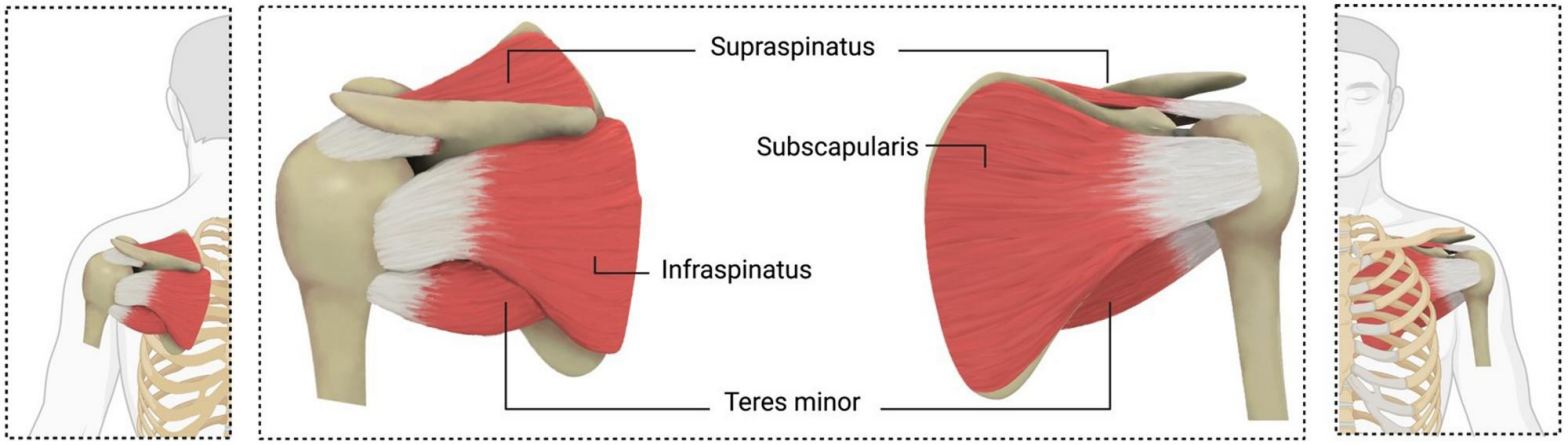
RC anatomy and the involved tendons including supraspinatus, infraspinatus, teres minor, and subscapularis.

The rotator cuff (RC) is a cuff of soft tissue responsible for the shoulder movement (Figure 1). Rotator cuff tears (RCTs) with more than 200 000 annual repair procedures in the United States are a significant cause of disability in adults [1–13]. Tendon detachment can cause tendon retraction, muscle degeneration, and shoulder instability [1, 14, 15]. One of the challenges with RC repair is the high retear rate after surgery. Patient age, tear size, repair strategies, and the degeneration of RC muscle influence the rate of retear [2, 16, 17].

RC muscle degeneration is characterized by atrophy of muscle fibers, fibrosis, and fat accumulation within and around the muscles [14, 18, 19]. Especially, fat accumulation of the muscle has a significant influence on the high rate of RC retear after surgery.

**Figure 2.**
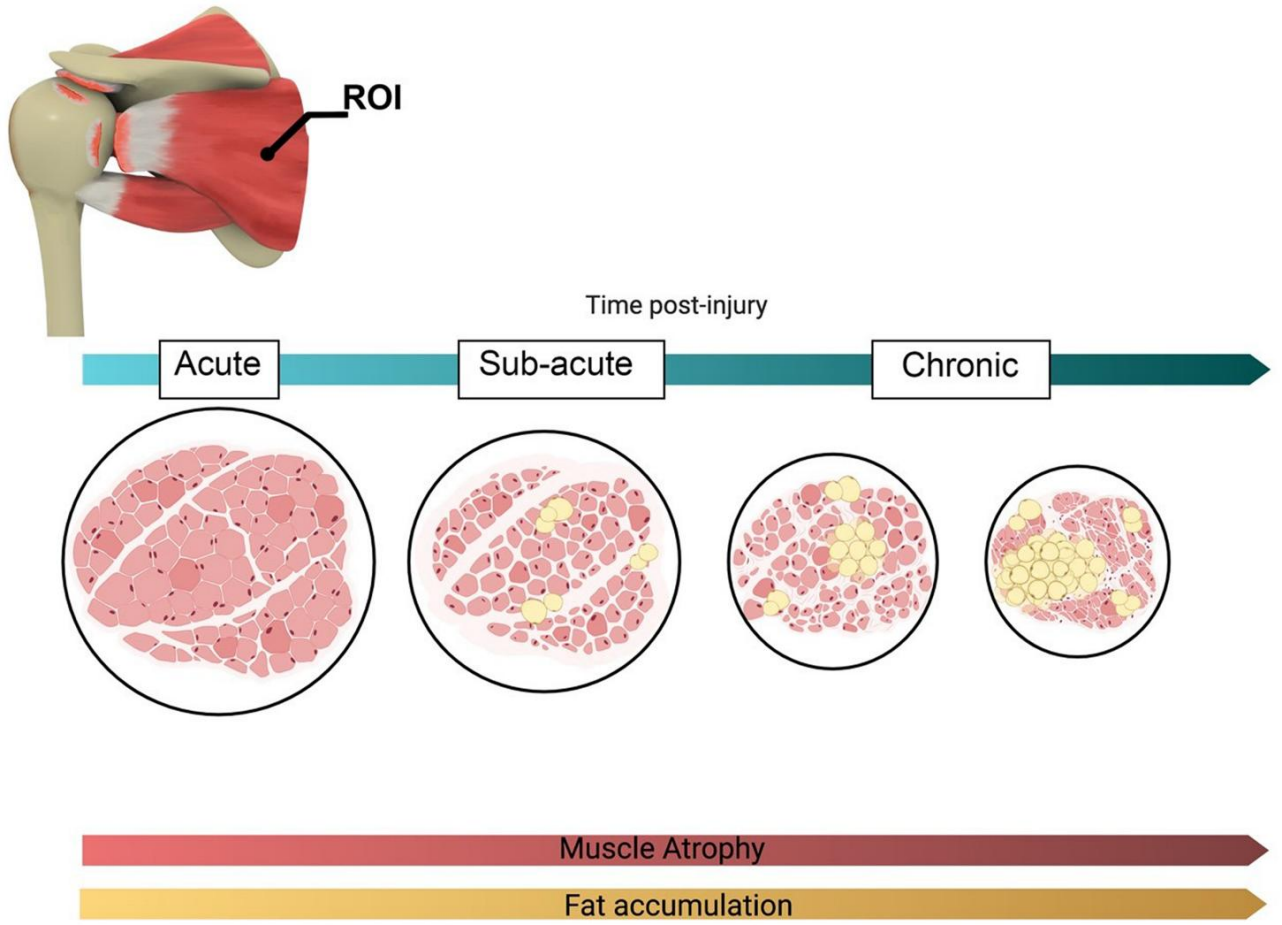
Muscle atrophy and fat accumulation of RC muscles in a Sub-acute and chronic injury (ROI, region of interest).

Clinically, the delay of surgical repair after RC injury is one of the most commonly observed challenges. This duration between injury and repair is described by category, ‘acute’, ‘subacute’ and ‘chronic’. An acute RC injury involves the immediate repair of the injury, while subacute and chronic refer to a prolonged duration. Subacute and chronic RCTs are often associated with a set of degenerative changes (Figure 2) [20, 21]. The tendon retraction and degenerative changes of the musculotendinous unit over weeks and months lead to muscle atrophy, fibrosis formation, and fat accumulation [19, 22–25].

The current surgical and nonsurgical strategies have failed to treat RCTs satisfactorily and tissue regeneration strategies are promising approaches to address the challenges [2, 26, 27]. Regenerative engineering stated as the convergence of disparate fields including stem cells science, advanced materials, physics, developmental biology, and clinical translation for the regeneration of various complex tissues, including musculoskeletal regeneration [1, 14, 19, 28].

Over the past two decades, significant progress has been made in the area of tissue engineering, which reveals a promising future for engineering complex tissues and organ systems [1, 14, 19, 29–34]. ‘Matrix’ or ‘Scaffold’ is a main component that plays an essential role in tissue regeneration, as it provides an important niche for cell attachment, proliferation, and differentiation.

For successful RC regeneration, materials need to be biodegradable and have appropriate physiological properties similar to the native tissue to provide a biomimetic substrate for cell activation [35, 36]. The materials and architecture that scaffolds can be fabricated from are determined based on the application of the scaffold in the body. Natural and synthetic polymers are a wide group of biomaterials that are used with different geometries for various kinds of applications [19, 29, 31, 37–43]. We have previously reported a comprehensive review of the use of natural and synthetic polymeric scaffolds for the regeneration of the RC [1].

Several strategies have been applied to stimulate cells and enhanced cell growth and activity for tissue regeneration [19, 29, 30, 33, 44–46]. One of the strategies for local stimulation are electroactive materials (EAMs). In the 1960s, Bassett *et al.* reported the efficiency of electrical stimulation (ES) on cells and tissues [47]. It has been reported that ES can rapidly affect cell migration and subsequently cell behavior [47]. Afterwards, several studies investigated the functionality of EAMs for tissue regenerative engineering. Electroactive-based materials have been widely used for the regeneration of many electrically excitable tissue types such as skeletal muscle [33], cardiac muscle [48], nerve [49], and bone [50] to enable the conduction of the natural electrical current along the bioactive graft after implantation *in vivo* [51]. ES has also been used in conjunction with such EAMs to recapitulate the *in vivo* electrical current passing through the native tissues to promote cell alignment, migration, proliferation, and differentiation to maintain the engineered tissue functionality [52, 53]. In this review, we first discuss the RC muscle degeneration and then review the applications of EAMs for the regeneration of skeletal muscle. In addition, we touch upon the utilization of ES with such EAMs.

## RC muscle degeneration

RC muscle degeneration is characterized by muscle atrophy, fibrosis formation, and the fat accumulation in the muscle. Clinically, these degenerative changes in a chronic RC injury are considered permanent changes that cannot be retreated. In this section, we review the current understanding of these changes and underlying mechanism for their appearance.

### RC muscle atrophy

Skeletal muscle forms almost half of the total body mass and comprises bundles of aligned muscle fibers [54, 55]. The growth and maintenance of muscle are supported by satellite cells which participate during the myogenesis process by their differentiation to myoblasts and subsequent fusion into myofibers [54–56]. The minor injuries of skeletal muscle are repaired by the intrinsic regeneration potential of satellite cells. However, this repair mechanism cannot support muscles in chronic RC injuries to inhibit or reduce fat accumulation and atrophy [14, 19]. Severe damage to muscle tissues prevents satellite cell recruitment and activation and limits regeneration [55].

The muscle atrophy is one of the critical factors that significantly affect the RC healing. It has been reported that muscle atrophy increases with aging even in healthy RC [57]. The number of satellite cells are specifically reduced in type II but not in type I skeletal muscle fibers in aged population [58]. However, the overall number of satellite cells did not change significantly with tear size [59], suggesting satellite cell niche is influenced by RCT. For example, muscle size and function are controlled by the balance of biogenesis/biosynthesis versus removal/destruction [60]. Insulin and insulin-like growth factor 1 are known to promote muscle growth, while TGF-β/myostatin/activin is known to prevent muscle growth [60]. In the RC, TGF-β signaling was upregulated followed by the RCT [61] and TGF-β expression was increased with age [62]. Another study showed the pro-myogenic gene expressions were suppressed in full-thickness tear and massive tear patients [63]. The ECM such as collagen type IV and laminin is another important regulator of skeletal muscle cell function and stem cell niche [64]. The remodeling of ECM followed by RCT may affect muscle regeneration through altered ECM environment [65]. The neuropathy of suprascapular nerve also influences to the progress of both muscle atrophy and fat accumulation [66].

Studies showed that the severe muscle atrophy makes the RC tendon irreparable [57, 67, 68]. RC muscle atrophy has been evaluated in several studies [19, 25, 67–70]. The common criteria to evaluate the muscle atrophy is muscle mass reduction, muscle fiber cross-sectional area, and muscle atrophy grading [19, 67–72]. The most common parameters for grading the muscle atrophy are the decreased muscle fiber size, a round shape of muscle fibers rather than an angular shape, and the decreased distance between myonuclei [68, 71, 72]. Several studies evaluated the different time-points of chronic injuries in different species, replicating the same muscle atrophy often seen clinically. Abdou *et al.* reported mild to moderate muscle atrophy from 2 to 6 weeks and severe atrophy at 8 and 12 weeks after injury in the supraspinatus muscle [68]. In a model developed by Kim *et al.* of chronic RCTs in rats, the extent of muscle degeneration after 16 weeks was found to be greater than that observed after 2 and 8 weeks [71]. Tang *et al.* [19], also reported muscle atrophy at 6 weeks following the RCT in a rat model.

### RC fibrosis

Fibrosis, also known as fibrotic scarring, is characterized by excessive synthesis, remodeling, and contraction of extracellular matrix [73]. In general, wound healing process contains four overlapping phases: hemostasis, inflammation, proliferation, and tissue remodeling [74, 75]. Chronic inflammation or inappropriate regulation of inflammation result in continuous ECM production that prevent tissue repair [76]. Several signal pathways, such as TGF-β, bone morphogenic protein, and Wingless/Int signaling, have been linked to the pathophysiology of fibrosis [73, 77, 78]. Myofibroblasts differentiated from fibroblasts in accordance with macrophage stimulation are characterized by the expression of collagen type I and α-smooth muscle actin (α-SMA). Various cytokines and growth factors including TGF-β are released from inflammatory cells, which regulate myofibroblasts growth and ECM synthesis [79, 80].

Tendons are composed of a highly organized ECM bundle such as predominantly collagen type I and subsequently collagen type III [81], which is responsible for transmitting generated forces from muscle to bone. Tenocytes which express tendon marker genes such as scleraxis (Scx) and tenomodulin (Tnmd) are predominant cell type in tendon and located between collagen fibrils. Tendon possesses less cellularity and vascularity compared to other tissues [82, 83], and very little turnover of ECM after maturation [84]. Upon tendon injury, various cell types including intrinsic and extrinsic cell sources are involved in its wound healing process [85]. However, adult tendons lack the inherent regeneration ability and result in fibrotic scar formation. Lineage tracing analysis of transgenic mice showed the contribution of Scx-lineage cells in neonatal healing whereas no recruitment into the defect area in adult [86]. Another lineage tracing study suggested αSMA progenitors contribute to newly formed tenocytes [87]. Without Scx-lineage cells, extrinsic cells seem to be recruited to the wound site and differentiate into myofibroblasts which is contributed to fibrosis formation. Even though tendon stem/progenitor cells in tendon have been identified [88], little is known about their contribution during tendon healing. The niche of TSCPs might be affected by complex process of wound healing or aging.

Clinically, tendon disease are classified from acute inflammation stage, chronic tendinopathy, and tendon rupture, causing shoulder pain [89]. Followed by RCT, torn tendon tissue increased cellularity and disorganized ECM content which leads fibrosis formation. In addition, intramuscular fibrosis formation plays a significant role in RC pathology [90]. After the RCT, synthesized intramuscular collagen content seemed to cause increased passive stiffness that is associated with degenerative changes [91]. In a clinical setting, immobilization of joint is important for tendon healing, but disuse of joint and muscular weakness can lead to shoulder stiffness [92, 93]. Although collagen is the central component of tendon healing, establishment of aligned bundle is essential to appropriate repair otherwise fibrosis formation losing original function.

### RC fat accumulation

**Figure 3.**
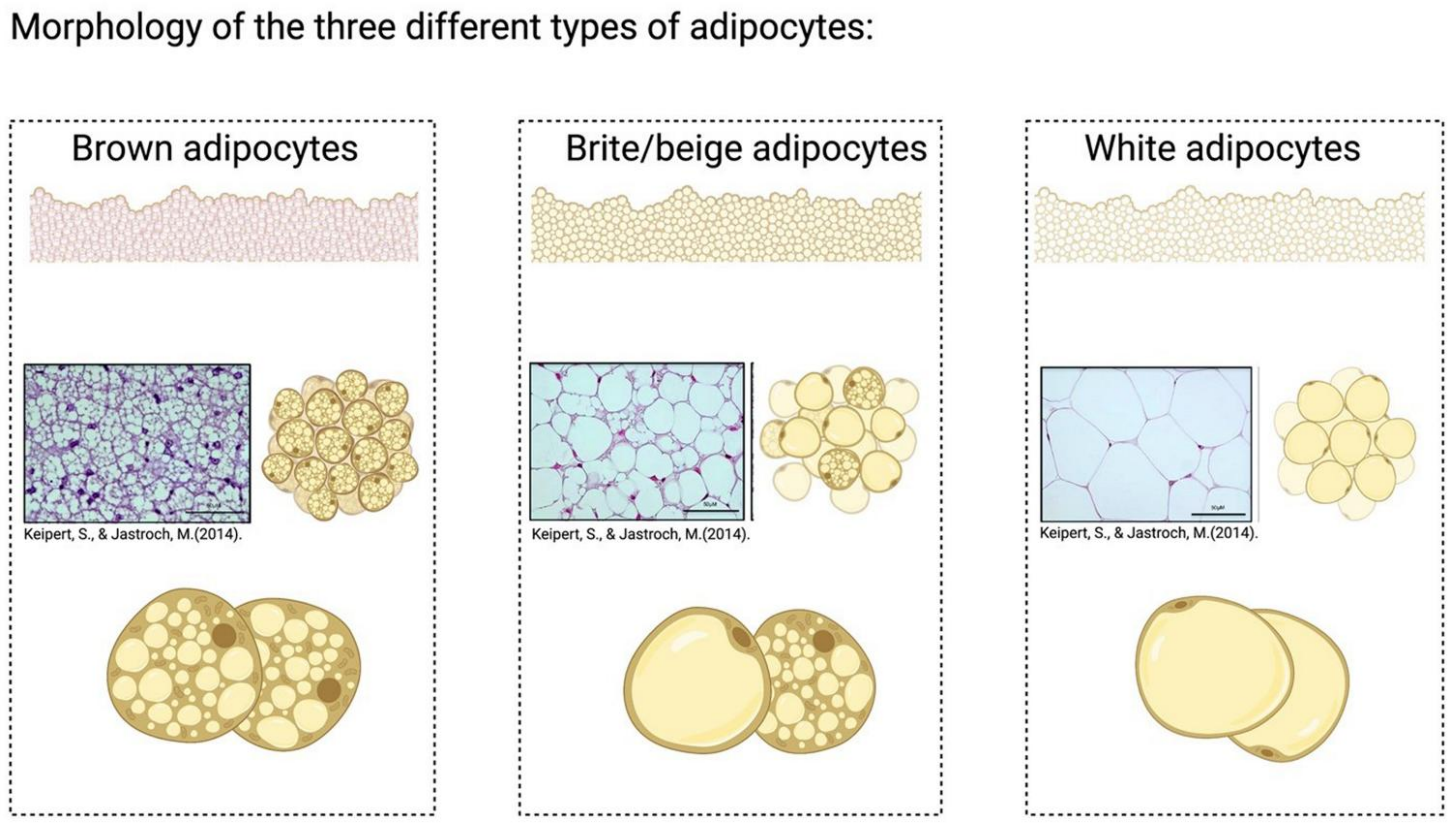
The schematic images of the morphology of different types of adipocytes include brown, brite/beige, and white adipocytes. The H&E histology images were published by [96].

In general, three types of adipose tissue have been identified by their anatomical location and metabolic features termed white adipose tissue, brown adipose tissue, and brite/beige adipose tissue (Figure 3) [94]. The white adipose tissue is the main energy-storing tissue and morphologically has one single big lipid droplet. On the other hand, brown adipose tissue’s responsibility is to dissipate energy as heat and is formed by many small lipid droplets. Despite white adipose tissue, the origin of brown adipose tissue is more closely related to muscle [95]. It derives from precursors that express Myf5 which is also expressed in myogenic lineages [96]. The expression of uncoupling protein 1 (UCP1) is a brown adipose tissue-specific marker. It has been reported that under specific stimulation, white adipocytes undergo a browning process which alters white adipose tissue into a brown adipose tissue phenotype, called beige adipocytes [94, 95, 97]. Brite/beige adipocytes form in white adipose tissue and show similarity to brown adipose tissue as they have multilocular lipid droplets and express UCP1, which distinguish them from white adipose tissue [96].

Fat accumulation is a more frequent challenge in RCTs compared with other musculotendinous injuries. Besides evaluating the effects of RCT, the effect of aging has also been reported on fat accumulation [57]. Many different cell types contribute to regenerate an efficient skeletal muscle. These cells can be either resident or not resident including but not limited to pericytes, fibro adipogenic progenitors (FAPs), muscle cells, and immune cells [98].

FAPs are muscle resident progenitor cells and were first described by Uezumi *et al.* [99]. These mesenchymal progenitors are positive for platelet-derived growth factor receptor alpha (PDGFRα). These cells are located in the muscle interstitium. The lack of paired box protein 7 (PAX7) expression distinguishes FAPs from muscle stem cells (satellite cells). It has been reported that these progenitors are the main source of fat accumulation and fibrosis in skeletal muscle [25, 65, 67, 99–102]. Lee *et al.* [101] demonstrated the intrinsic differences in the distribution and differentiation of FAPs in RC muscle compared with muscles from other anatomical locations in mice [101]. Supraspinatus and infraspinatus muscles showed the highest percentage of progenitors (17.5% and 15.7%, respectively) compared with tibialis anterior muscle (5.3%) based on fluorescence-activated cell sorting [101]. The highest concentration and adipogenic potential of FAPs in RC muscles can be the most important contributing factors in fat accumulation after RCTs. Clinically it has been reported that supraspinatus in a chronic RCT has a great number of FAPs and fibrosis compared with healthy RC [103]. Also, it has been shown that increasing the RC tear size and thickness leads to an increase in the number of FAPs [59].

Initially, after muscle injury, FAPs start to proliferate and provide redifferentiation signal for myogenesis which is beneficial for muscle regeneration [65]. After the initial expansion, a large number of progenitors undergo apoptosis. However, some of them persist and form the main source of fat accumulation and fibrosis [65, 98]. Although the exact underlying mechanism by which FAPs undergo adipogenesis and fibrogenesis is not identified yet [65].

The proliferation and differentiation of satellite cells depend on the composition of the niche and ECM signaling. The fat accumulation and fibrosis formation by FAPs can change the ECM composition which subsequently alters satellite cells pathways and can negatively affect their regenerative efficacy [65]. It has been reported that FAPs may also express anti-myogenic signals such as TGF-β [65].

Several studies reported an increase in the severity of fat accumulation by time in chronic RCT models [71]. The severity of fat accumulation is commonly reported by grading or fat area calculation [67, 68, 71,72, 104–106]. Goutallier *et al.* [107, 108], described a standard system to classify the severity of fat accumulation after RCTs. Clinically, stage 3 and stage 4 of Goutallier grading of the torn RC is considered irreparable while stage 1 or stage 2 may be reversed by an ideal treatment of fat accumulation [68, 107–109]. The developed strategies to stop, reduce, or reverse the fat accumulation following the RCTs will be discussed further in this article.

## Electroactivity as a novel strategy to reverse the muscle degeneration after RCTs

Several strategies to treat RCT have been utilized and evaluated. These include various suture-based techniques such as a single row, and double row [110, 111], the use of tissue transplants (allograft, xenograft, autograft, decellularized structures), and tissue regeneration strategies [2]. The current standard treatment for muscle regeneration is to engraft healthy autologous tissue from an uninjured site [56]. Although successful, almost 10% of autograft transplantation fails due to infection and necrosis [112, 113]. Moreover, autografts present limitations such as donor morbidity and limited availability [114]. These limitations have driven efforts to develop alternative strategies to support muscle regeneration. Clinically, current surgical practices, including suture techniques and tissue transplants, have failed to treat RCTs successfully. It has been reported that the current strategies cannot reverse muscle degeneration after RC repair [19, 115–117]. There is still a need to reduce fat accumulation and muscle atrophy after RCT to improve RC regeneration. One of the most novel strategies is the applications of EAMs for reducing the muscle degeneration.

The human body is known for its ability to generates endogenous electrical potential, referred to as bio-electricity, that plays a key role in the homeostasis of various tissue types, including tissue development and wound healing [118]. When an injury occurs, an injury potential is initiated as a steady direct current (DC) electric field (EF) that localizes at the injury site. This localized EF guides cells to migrate and sprout directly toward the wound edge to aid in the healing process. Without EF, the wound healing is inhibited [119]. During cell migration to the wound edge, a voltage gradient of action potentials is initiated across the cell membrane as a result of the EF, causing the cell membrane to depolarize. This triggers cells to transmit biomolecular signals and upregulate certain genes and hormones that are essential during the healing process [119].

In some cases, some parts of the body such as tissues or organ systems lose their ability to endogenously generate an action potential EF or generates very weak action potential that cannot initiate a steady EF to perform the natural bioelectrical operations. However, an action potential can now be exogenously elicited by inducing an electrical charge to the cells, mimicking those of the endogenously produced through a process termed ‘electrical stimulation’ [120].

Electroconductive biomaterials can serve as a critical cue to promote muscle regeneration by improving the interactions between materials and cells [27, 121]. Since skeletal muscle is an electrically excitable tissue [27, 32, 121], the implantation of EAMs can support myoblast growth and maturation [27, 36, 121, 122]. Several studies indicated that EAMs could significantly promote myoblasts’ viability and differentiation, even with no external ES [19, 27, 32, 121, 123]. The efficacy of EAMs such as graphene [27, 124], and conductive polymers including poly(3,4-ethylene dioxythiophene) (PEDOT) [19, 32], polypyrrole (PPy) [125], and polyaniline (PANi) [121] has been reported *in vitro* and *in vivo* for skeletal muscle regeneration [121, 126–128].

### External electrical stimulation

ES is a process used to provide an exogenous action potential to the cells, in the absence of the endogenous action potential [120]. In 1942, ES was proposed as a useful technique to preserve muscle tissue functions by substituting the nervous stimulation in denervated skeletal muscles [129]. The mass and contractility of denervated muscle tissues were maintained and improved by this technique [129]. Since then, ES has extensively been applied in both, clinical settings and tissue engineering as an alternate to treat various muscular and neuronal diseases, improve blood circulation at diseased tissues, promote wound healing, and maintain the functionality of the engineered tissues in culture [130]. The ability of various biological systems to respond to exogenous action potential suggests that ES may serve as an efficient tool to control and adjust cellular and tissue homeostasis [131, 132]. The responses of different types of cells to ES have been studied, including mesenchymal stem cells (MSCs), fibroblasts, osteoblasts, myoblasts, dorsal root ganglia, and neural crest cells [133–138]. These studies have shown that ES affects important cellular behaviors such as adhesion, proliferation, differentiation, directional migration, as well as cell division, all of which are essential elements during the tissue development and wound healing process. The variation in tissue composition, such as tissue type and density, cell membrane permeability, and electrolyte leads to the variation in the electrical resistivity of biological tissues. Therefore, the method by which the ES is applied must be optimized as a function of cell type and tissue composition to achieve the desired therapeutic effects [139].

#### Different methods of ES

ES is a feasible technique for both *in vivo* and *in vitro* two-dimensional (2D) and three-dimensional (3D) cultured cells [140]. Although methods for ES vary, no specific procedure/methodology/setup has been standardized for every cell or tissue type. Based on the literatures, ES methods can be characterized into: (i) DC/alternating current EF (AC EF), (ii) capacitive coupling EF (CC EF), and (iii) inductive coupling EF (IC EF) [139]. Various cell types have been studied and found to be responsive to these ES methods under different experimental settings [133–138].

##### Direct current/alternating current EF

ES was considered as a new therapeutic substitute and studied using both DC and AC EF following the finding of ossification enhancement by DC EF [141]. In medical literatures, DC and AC EFs refer to either electrical potential gradient or electrical current between two points of different electrical potentials generated by a power source [139]. DC/AC EF can be generated between an anode (−) and a cathode (+) immersed in electrolytes. In biological application, this electrolyte can be culture medium or tissue fluid and the current carriers are the ions in it. The current passing between the electrodes can cause depolarization of the membrane. Depending on certain parameters such as the EF frequency, current, and voltage, different degrees of depolarization can be obtained to achieve different experimental outcomes [142]. Electrodes can be metallic such as stainless steel, tungsten, platinum, platinum iridium alloys, iridium oxide, and titanium nitride; carbon-based such as carbon nanotubes (CNT); or made from conductive polymers such as PPy [139]. These electrodes can be simply altered into different forms and sizes, connected with a power source, and immersed directly into culture medium or tissue [139]. This procedure to apply ES to the cells is relatively simple; however, it has some disadvantages. The conversion of ionic conductivity into electron conductivity in these electrodes generates electrochemical reactions on the electrode surface which forms chemical species (faradic products) [139]. The faradic products in most cases lead to lowering of the media calcium levels, gas formation, and significantly increase the pH levels in the culture medium [143]. In addition, the direct exposure of the electrodes into the culture medium can cause disruption for the electrolyte composition when the electrical potential is applied and the current passes. Moreover, toxicity in the culture medium can arise, due to the insufficient biocompatibility of some electrodes, causing reduced levels of molecular oxygen which further hinder the use of this method [144, 145].

##### Capacitive coupling EF

Compared to the DC/AC EF method, CC EF offers a safer way for delivering the electrical stimulus to the cultured cells. In CC EF, a homogenous electromagnetic field is created between two parallel layers of metallic electrodes (e.g., stainless steel, gold) or carbon-based electrodes (i.e., a plate capacitor) that are placed above and below, the cell culture plate with a small (0.5–2 mm) gap between them [146–157]. CC EF has the advantage of generating a homogenous EF which leads to the equal amount of stimulation for every cell regardless of their position in the culture vessel. The main disadvantage of this method is the high voltage (e.g. 100 V) that is required between the electrodes [139].

##### Inductive coupling EF

In the IC EF, controlled electromagnetic fields are generated by coils placed around the cell culture plate. Compared to the CC EF method, IC EF has the advantage of generating small order of magnitude of currents and potentials near the targeted cells, rather than delivering the stimulation through electrodes [149, 152, 158–161]. Here, the coils are used in pairs, placed in a Helmholtz configuration, with a distance between the coils equal to their radius [162–164]. This method generates a homogenous magnetic field across the cell culture plate with uniform electromagnetic field properties. One of the subcategories of this method is pulsed electromagnetic field (PEMF) stimulation. In PEMF, the stimulus is delivered in pulses rather than being static or continuously harmonic [162, 165].

### Electroconductive materials

The methodologies discussed in the previous section have been extensively used for exogenously delivering the electrical stimulus to the cultured cells, as well as to study the cell response to the ES. However, they can be problematic when applied for tissue regeneration applications, especially when a porous (3D) scaffold is used in culture. Since most fabricated scaffolds/matrices are not electrically conductive (dielectric), cells inside the porous scaffold or on its surface are not exposed to the same degree of EF and current. Therefore, this can generate a heterogeneous cell population of electrically stimulated and non-stimulated cells, which can lead to unmet experimental outcomes [139, 166].

Significant effort has been made in the past two decades for developing innovative strategies for the fabrication of biomimetic scaffolds for tissue regeneration [167, 168]. These efforts enabled the fabrication of scaffolds in a verity of physical forms such as fibers, meshes, membranes, gels or solids [167]. Of these, fibrous-based scaffolds have gained much attention and are particularly suitable for tissue regeneration applications due to their structural and topographical resemblance to the natural ECM [1, 14, 29, 32, 33, 169]. Fibers can be fabricated in the nanoscale/microscale by applying different fabrication techniques such as electrospinning, self-assembly, phase separation, and template melt-extrusion [32, 167, 170–173]. Moreover, fibers can be processed into complex fibrous structures using various textile techniques, such as knitting, weaving, or braiding, to create 3D structures with improved structural/mechanical properties [169]. It is nevertheless still a challenge to develop bioactive fibrous-based biomaterials that can enhance cell proliferation and guide their differentiation. Although, fibers can be modified either chemically or physically to show appropriate physiochemical and biological properties similar to natural niche environment to enhance cell adhesion, proliferation, and differentiation and ultimately improve tissue regeneration [51]. EAMs such as conductive polymers, electrets, piezoelectric and photovoltaic materials have been widely investigated for tissue regeneration applications due to their intrinsic chemical and physical properties, as well as high electrical conductivity that can modulate cellular response [174–181]. Due to nonbiodegradability and poor processability of such materials, they are often used in conjunction with other synthetic or natural biomaterials as composites, surface coatings, or functionalized on the surface of other materials via *in situ* polymerization technique [51, 182]. The electrical activity of these materials can be measured using conductivity measuring devices [183] and also other methods have been used to detect the composition and the dopant, functional groups, or coatings added to them such as UV-vis, Fourier transform infrared spectroscopy, elemental microanalysis, X-ray photoelectron spectroscopy, differential scanning calorimeter, and thermogravimetric analysis [184] in some studies researchers have measured the electrical resistivity of conductive polymers by four-point probe method [184–187]. Also, the characterization of PANI using dielectric relaxation spectroscopy and electron paramagnetic resonance has been done to study the electrical properties of polystyrene/PANI powder which is then compressed to form pellets [188]. The versatility of fibrous-based structures allows for combining such EAMs in their structure to form electro-active-based scaffolds [51].

EAMs include carbon-based composite, and conductive polymers can provide high similarity with the physiological properties of native tissues. EAMs intrinsic properties of simultaneously displaying the physical and chemical properties of polymers and the electrical characteristics of metals, all of which are key properties for regulating cellular functions, including differentiation, adhesion, proliferation, and migration [189]. Not only EAMs would allow for homogenous delivery of exogenous ES to the cultured cells *in vitro* but would also allow for homogenous delivery of endogenous ES along the bioactive scaffold after implantation *in vivo*. Studies have shown the potential of EAMs to enhance cellular activities, including cell adhesion, migration, proliferation, differentiation, and protein secretion with or without ES [125, 190–195]. This however, suggests the strong potential for such bioactive materials for use as suitable platforms for the regeneration of varies tissue types, especially of electrically excitable tissues such as skeletal muscles, cardiac muscles, nerves, connective tissues and bones [196, 197].

#### Conductive polymer

Among all types of organic and inorganic conductive biomaterials, PPy, PANi, and PEDOT are the most popular EAMs for tissue regeneration [36, 198]. Here, we briefly review the general properties of these conductive polymers.

##### Polypyrrole

Conjugated PPy is one of the most investigated conductive polymers because of its high electrical conductivity and good chemical stability in air and water and most importantly, good biocompatibility [199–201]. The polymerized PPy has a rigid conjugated backbone caused its insolubility in solvents and therefore limited processability. However, the monomer pyrrole is soluble in various solvents. To overcome this limitation, researchers incorporate the monomer pyrrole before polymerization into the matrices. It is difficult to fabricate pure PPy matrix due to its rigidness, brittleness, and nonbiodegradability [126, 194]. PPy can be polymerized in aqueous solution by various oxidizing agents such as FeCl_3_, (NH_4_)_2_S_2_O_8_, CuCl_2_. (NH_4_)_2_S_2_O_8_ is used for higher speed reactions (a few minutes) while FeCl_3_ is for lower speeds (e.g. 6 h) [202].Also, solvent-free oxidative polymerization methods have been used to mechanically mix the monomer and solid oxidant [183]. Due to the nonbiodegradability of PPy, it is important to use the lowest concentrations of PPy to ensure the biocompatibility of the PPy-matrix upon degradation [203, 204]. It has been reported that a small amount of PPy in the polymeric matrices such as poly(dl-lactic acid), poly(l-lactic acid) (PLLA), and polycaprolactone (PCL), has no adverse effects on their degradation behavior [205].

##### Polyaniline

PANi can be polymerized by different chemical and electrochemical oxidative methods. There are various structures of PANi based on the oxidation states; pernigraniline base (the fully oxidized structure), emeraldine base (the half-oxidized structure) and leucoemeraldine (the fully reduced structure) [201, 206, 207]. Besides the advantages of PANi including good conductivity, low cost, and good stability [208, 209], there are some challenges which limit its biological application. Similar to polymerized Ppy, PANi has poor processability, nonbiodegradability, and more importantly, it has been reported that it can cause chronic inflammation after implantation [210–213].

##### Poly(3,4-ethyl-enedioxythiophene)

PEDOT is another conductive polymer that is being investigated for tissue regeneration and is one of the polythiophene derivatives. PEDOT is a conjugated polymer which shows desirable chemical, electrical, and environmental stability and also better thermal stability and conductivity compared to PPy [214, 215].

Poor solubility is one of the critical challenges in using PEDOT which leads to poor processability. However, PEDOT with polystyrene sulfonate (PSS) composition shows improved solubility [216]. PEDOT could also be a promising conductor for biomedical applications. The good biocompatibility and low cytotoxicity of PEDOT have been reported in several *in vitro* and *in vivo* studies. Similar to other discussed conductive polymers, high stiffness and nonbiodegradability of PEDOT make some limitations which need to be addressed by keeping its concentration as low as possible [217].

#### Carbon-based materials

Carbon-based composites such as graphene, diamond, CNT, and nanowires have been investigated in different applications include electronic devices, drug delivery and tissue regenerative engineering [175, 218].

Graphene has been investigated for several biomedical and medicine applications owing to its unique features including the high electrical conductivity, high mechanical strength, stability in aqueous environments, ease of functionalization, flat structure with a large surface to volume ratio, heat conduction, and optical transparency [124, 219, 220].

A single atomic layer of sp2-hybridized carbon atoms (two single bonds and one double bond) organized in a hexagonal lattice is the base structure of graphene materials [219, 221]. The three covalent sigma (σ) bonds (the strongest type of covalent chemical bonds) among the carbon atoms form the sp2 configuration. This means a free valence electron is left which participates in a π bond that emerges from the lattice in the *z*-direction. The delocalized electrons in the π orbital give graphene its unique electronic and physical properties. The graphene’s inherent electrical conductivity can create electrical coupling with cells which may change the electrical activity of the cells [219, 221]. The formation of noncovalent interactions such as π–π, hydrogen bonding, and electrostatic between graphene nanomaterials and biomolecules can be considered as a contributing factor affecting cell differentiation.

Several studies reported the ability of various forms of graphene to support cell growth and induce differentiation in different cell lineages such as bone, neural, cardiac, and skeletal muscle [124, 219, 220, 222].

### Mechanism of electrical field

Several studies demonstrated that applying ES on conductive polymers improve the cell activities and growth. Indeed, the conductive polymers with both electrical and ionic charges by inducing new conducting pathways/channels, help the cells communication together [36]. The mechanisms by which ES enhance the cells differentiation are still unknown. Wang *et al.* [223] reported a higher concentration of calcium ions in cardiac myoblasts on an EAMs-based scaffold. Under the condition with no external ES, the calcium ions concentration was higher on the conductive scaffold compared to the control group [223].

Calcium is one of the important intracellular messengers in myocytes. It plays an important role in muscle excitation–contraction and modulation of gene expression during myogenesis [224]. During the contraction, a voltage-gated L-type calcium channel is opened which allows the transport of small quantities of calcium ions into the cells. These calcium ions trigger the sarcoplasmic reticulum to release more calcium ions [224]. A large amount of intracellular calcium ions activates the myosin cross-bridge cycling which leads to muscle contraction [224]. Besides this direct activation, calcium ions can also indirectly induce the contraction through calcium/calmodulin-dependent protein kinase II and other enzymes and proteins involved in muscle contraction [224]. The concentration of calcium ions in the mitochondrial and nuclear matrices is similar to the calcium ion levels in the cytoplasm [225]. The increase in the cytoplasmic calcium ion levels can be attributed to various stimulations and result from either the release of calcium ions from internal stores or the influx of extracellular calcium ions by the calcium ion channels [225]. It has been reported that intracellular calcium ions alter the muscle gene’s expression and subsequently myogenesis. Porter *et al.* [224] showed that the presence of L-type calcium channel blockers inhibited the differentiation of C2C12 myoblasts to form myotubes. The study also reported that lowering the intracellular calcium ions through Dantrolene and Thapsigargin inhibited myotube formation and maturation (Dantrolene blocks calcium ions release from the sarcoplasmic reticulum and Thapsigargin inhibits the entry of calcium ions into the sarcoplasmic reticulum) [224].

Zhang *et al.* investigated the efficacy and the mechanisms of ES on PPy-based scaffold seeded with adipose-derived mesenchymal stem cells (AD-MSCs) [44]. Incorporation of PPy with the content of 9.6% increased the mechanical properties, alignment of cells, and hydrophilicity and subsequently the penetration of cells to the deeper/inner region. A 200 μA of direct current for 4 h per day for 3 weeks were applied on the cells. Based on the results, incorporation of EAMs with ES remarkably improved AD-MSCs migration and penetration. The significant enhancement of ALP activity, calcium deposition, and gene expression of osteogenic transcription factors confirmed the high potential of this strategy for bone tissue engineering. Further, different voltage-gated ion channels’ (VGIC) blockers (Ca2+, Na+, K+, and Cl− VGICs) were added to the AD-MSCs-seeded scaffolds. The data showed the key role of all VGICs and particularly Ca2+ VGIC on AD-MSCs migration and osteogenic differentiation [44]. The study indicated that applying ES to AD-MSCs seeded on conductive scaffold can change the membrane potential and activate the Ca2+, K+, Na+, and Cl− VGICs and subsequently alters the relevant ions flow among these VGICs to improve cell functions [44].

### Applications of electroconductivity skeletal muscle regeneration

One of the most abundant tissues in our body is skeletal muscle [226]. Upon injury, skeletal muscle can regenerate the lost tissue but to a certain threshold [227]. However, if the injury is severe, the muscle tissue is unable to entirely regenerate its function [114, 228, 229]. Regenerative engineering approaches may be able to regenerate or restore the function of the impaired muscle tissue using biomaterial scaffolds, which attempt to mimic the ECM of the native muscle tissue. Due to the structural and topographical resemblance of fibrous scaffolds to the natural ECM, they have been tested as possible regenerative potential for muscle cells.

The aligned conductive fibers promote the myoblasts’ differentiation into myocytes and support the fusion of myocytes together to form the myotubes. The maturation of these myotubes can lead to the regeneration of skeletal muscle [1, 230].

Jun *et al.* developed an electroactive randomly aligned composite fibers made from poly(l-lactide-co-epsilon-caprolactone) (PLCL)/PANi using an electrospinning method and investigated the effect of the composite on myoblasts viability, proliferation, and differentiation. The authors showed that the PLCL/PANi fibers group was biocompatible and an enhanced number of cells were positive for sarcomeric myosin compared to the PLCL fibers group. In addition, PLCL/PANi fibers also improved myogenin expression when compared to the PLCL fibers group. The gene expression of troponin T and myosin heavy chain were also enhanced by the conductive PLCL/PANi fibers, indicating that electroactive fibrous structure has synergetic effects on modulating myoblasts differentiation and fusion into myotubes even without the addition of ES [128].

The aligned anisotropic structure of elongated myofibers in skeletal muscle suggests that providing aligned components to the engineered scaffold may serve as a guidance to myoblasts alignment and formation into myotubes. It was found that the aligned underlying orientation of electrospun scaffolds could serve as topographical cues that enhanced the alignment and elongation of skeletal muscle cells [127]. Based on these early findings, Chen *et al.* developed an electroactive PCL/PANi aligned and randomly aligned electrospun nanofiber scaffolds to study the effect of the fibers’ alignment on myoblasts response. Immunofluorescence staining data revealed that the PCL/PANi nanofibers with highly aligned structure could guide myoblast orientation and promote myotube formation better than random fibers. In addition, the electroactive and aligned PCL/PANi nanofibers showed further enhanced myotube maturation when compared to all the other groups (electroactive randomly aligned PCL/PANi, nonelectroactive aligned, and randomly aligned PCL alone), demonstrating the synergetic effect of the topographical and electroactive cues together on the differentiation of myoblasts [121].

Beside the underlying orientation of the fibrous scaffold, the concentration as well as the strategy of which EAMs are introduced to fibrous scaffolds can also affect the response of the cultured myoblasts. It has been reported that increasing conductivity via increasing the concentration of the EAMs, can improve the myoblasts attachment, proliferation, and differentiation [231]. However, increasing the concentration of the EAMs usually increases scaffold stiffness, which may be detrimental to myoblast development [231]. For example, Tang *et al.* developed aligned electrospun PCL/PEDOT: PSS electroactive scaffolds coated with different PEDOT: PSS concentrations. In this study, myoblasts viability, proliferation, and differentiation cultured on the electroactive scaffolds with the different PEDOT: PSS concentrations (from 1% to 100%) were assessed. Although all electroactive scaffolds were shown to be biocompatible, it was found that low and medium coating concentrations (1% and 10%) showed stimulatory effect on myoblasts growth and differentiation, as increasing the concentration of the EAMs was shown to suppress the growth and differentiation of the cultured myoblasts. Despite the improved outcomes from culturing myoblasts on the low and medium PEDOT: PSS-containing scaffolds, scaffolds with higher amounts of PEDOT: PSS had the highest electrical conductivity [32]. However, higher concentrations of PEDOT: PSS may have also increased the scaffold stiffness, which caused suppression in myoblasts bioactivities.

Browe *et al.* developed an aligned electrospun PPy/PCL scaffold with different PPy concentrations for muscle regeneration. In this study, both PPy and PCL were synthesized together into a copolymer (PPy/PCL), which was designed to increase scaffolds conductivity without significantly influencing their stiffness. The scaffolds were characterized *in vitro* and assessed for their suitability for myoblast proliferation and differentiation. *In vitro* data revealed that the 40% PPy/PCL group had the highest electrical conductivity when compared to all the other groups (pure PCL, 10% PPy/PCL, 20% PPy/PCL), and that increasing the PPy concentration had no significant effect on the stiffness of the scaffolds. The results of fusion index and number of nuclei per myotube showed promoted myoblast differentiation on PPy/PCL than PCL. It indicates that the method of which the EAM is introduced to the polymeric scaffold may play a key role in altering the stiffness and modulating myoblasts response [231]. As indicated here, different EAMs have different mechanical and conductive properties. Thus, these studies suggest that the strategy in which the EAMs are introduced to the fibrous scaffolds must be optimized as a function of a conductive material type to achieve the best possible scaffold stiffness and conductivity without suppressing the potential for myoblasts proliferation, differentiation and ultimately muscle regeneration.

ES is another factor contributing to the differentiation of myoblasts to myotubes. It has been shown that myoblasts are able to differentiate into myotube when cultured on electroactive fibrous-based scaffolds, even in the absence of ES [32, 121, 231]. However, the presence of ES further enhances myotube formation and muscle contractibility [127]. Several works have utilized ES along on electroactive fibrous-based scaffold for further enhancing the functionality of the formed myotubes in culture [232, 233]. For electrically stimulating muscle myoblasts, the ES parameters are often optimized as a function of scaffold material, as myoblasts cultured on different EAMs response differently to the applied ES due to the variation of the electrical conductivity of these materials.

Quigley *et al.* developed an aligned fibrous-based scaffold made from CNT and PPy as a potential myogenic scaffold and investigated the ES effects on myoblasts proliferation and differentiation. In this study, the cultured myoblasts were electrically stimulated with (0.125 mA cm^−2^) at a frequency of (10 Hz) for 8 h per 24 h period for 3 days. It was found that myoblasts orientation, length, and size were significantly enhanced when they were electrically stimulated on the electroactive fibrous scaffold when compared to the non-electrically stimulated controls. In addition, ES significantly increased myoblasts proliferation and differentiation into myotubes [232].

Sirivisoot *et al.* further investigated the effects of ES on myoblasts response by fabricating a multiwalled CNT/PU electrospun scaffold. In this study, the cultured myoblasts were electrically stimulated with (22 V/cm) at a frequency of (20 Hz) twice a day on the last 2 days of culture (4 days total). Immunofluorescence staining data revealed that after ES, the number of multinucleated myotubes on the electrospun CNT/PU scaffolds was significantly larger than that on nonelectrically stimulated CNT/PU scaffolds. In addition, myoblasts were able to differentiate into myotubes on both scaffolds groups, but the expression of Myf5 and myosin heavy chains was significantly greater on the electrically stimulated CNT/PU group [233]. These studies demonstrate that electroactive platforms can be utilized to influence muscle cell behaviors through fibrous structures and ES. In addition, the data clearly indicate that ES parameters are dependent on the type of the EAM used in the scaffold, as the ES parameters in these studies varied when different EAMs with different conductivities were used. Thus, optimizing the ES parameters for myoblasts cultured on electroactive fibrous-based scaffolds is crucial for integrative guidance to the cultured cells.

To the best of our knowledge, few studies have examined the efficacy of EAMs for muscle regeneration *in vivo*.

Zhao *et al.* [234], showed the potential of a conductive cryogel for skeletal muscle tissue regeneration. The cryogel was fabricated by cryopolymerization of gelatin (GT) and poly(tannic acid) reduced graphene oxide (GT/rGO@PTA). The *in vitro* study using C2C12 myoblasts demonstrated the enhanced cell proliferation and myogenic differentiation with cryogel. Thereafter, the cryogel was evaluated in a rat tibialis anterior volumetric muscle loss model. The samples were harvested at 2 and 5 weeks of implantation. At 2 weeks, the histology of muscles showed new tissue formation in cryogel implanted groups compared with the control group. After 5 weeks, the muscle formation was detected in the cryogel-implanted animals with the highest centronucleated myofibers and capillaries compared with control groups. Moreover, the trichrome staining showed that the blank group had the most fibrosis formation than GT/rGO@PTA group. These results demonstrated that the EAMs-based cryogel promoted the regeneration of skeletal muscle [234]. The potential of GT and polydopamine-coated carbon nanotubes (PCNTs) cryogel was also investigated by Hu *et al.* [235]. Both *in vitro* and *in vivo* results showed the efficacy of EAMs-based implantation for muscle regeneration. The fabricated cryogel promoted the myogenic differentiation of C2C12 cells and new muscle formation in a rat tibialis anterior muscle defect model [235].

Du *et al.* [27] developed poly (citric acid-octanediol-polyethylene glycol) (PCE)-graphene (PCEG) nanocomposites. The *in vitro* assays showed enhanced C2C12 myoblasts proliferation and myogenic differentiation on PCEG nanocomposite. The efficacy of the PCEG nanocomposite was further evaluated in a rat tibialis anterior muscle injury. The histological results showed the formation of new muscle tissue at 1 week and the new tissue significantly increased at 4 weeks in the nanocomposite implanted group. Besides they reported high numbers of centronucleated myofibers and capillaries in PCEG group compared with the control groups after 1 and 4 weeks [27].

Tang *et al.* reported the efficacy of EAMs-based aligned fibers to retain muscle quality and enhance muscle regeneration after RCT in rat acute and subacute models [19, 126]. The scaffold was fabricated by coating PEDOT: PSS on PCL nanofibers. The regenerative potential of the scaffold was evaluated using two repair models of RCTs include acute and subacute [19, 126]. For the acute model, the scaffold was implanted on the supraspinatus muscle immediately after the injury. The repair surgery for the sub-acute model was conducted 6 weeks after injury.

**Figure 4.**
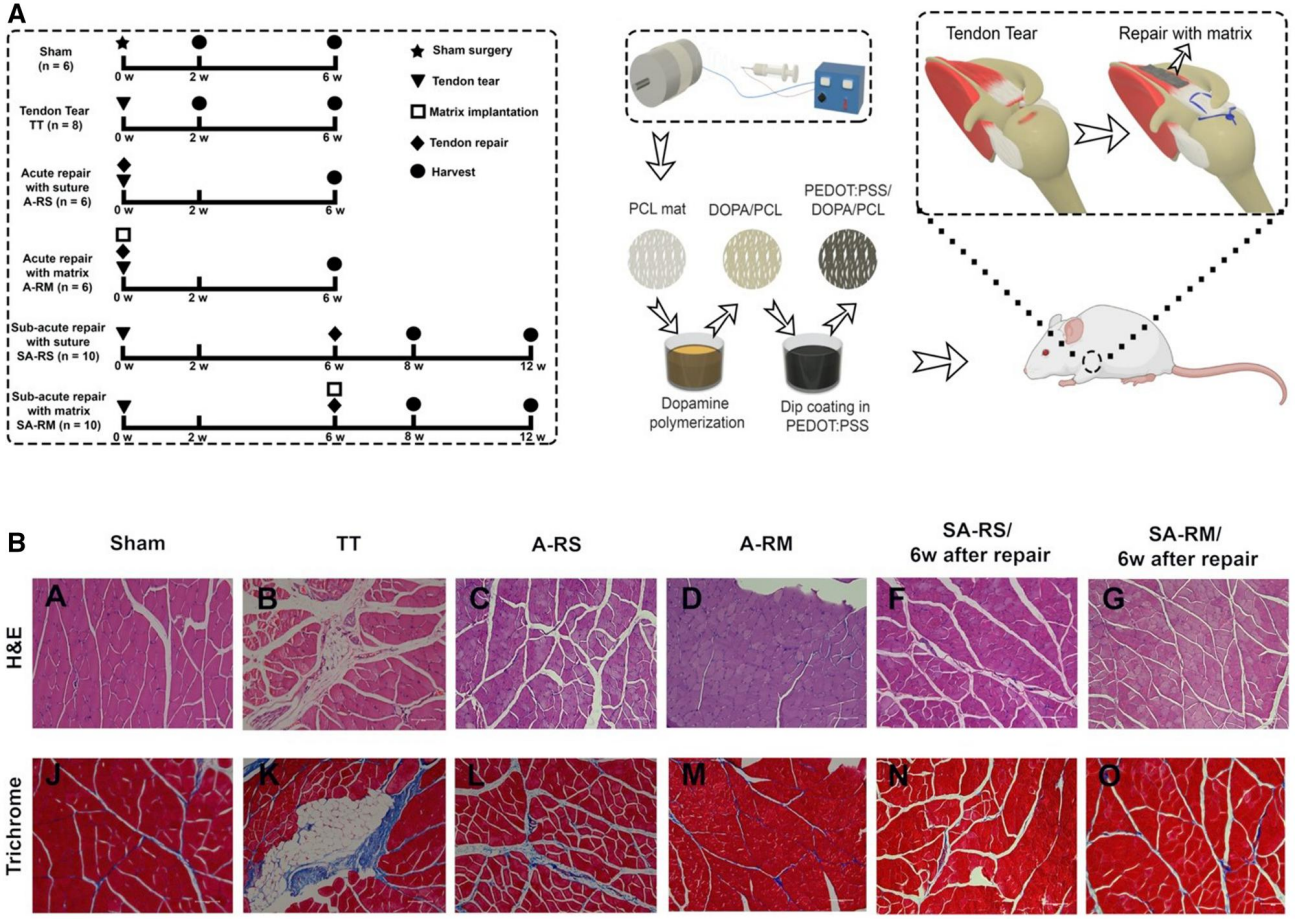
(**A**) Diagram of experimental groups and schematic illustration of the RC surgery model. For subacute groups, 4 and 6 rats were sacrificed 2 and 6 weeks after repair respectively. (**B**) Histology images of the supraspinatus muscle for both acute and subacute groups, (A) H&E, and (B) Masson trichrome staining [19, 126].

The results demonstrated the ability of aligned electroconductive fibrous scaffolds to reduce muscle atrophy 2 and 6 weeks after RC injury [19, 126]. Figure 4 shows H&E and trichrome staining of the rat supraspinatus muscle after 6 weeks of repair. The treated groups (A-RM and SA-RM/6w) showed better muscle organization 6 weeks after successful surgical repair [19, 126].

The results demonstrated the ability of electroconductive scaffolds to guide and stimulate muscle regeneration and treat muscle atrophy in RC injury. The control group showed muscle degeneration and atrophy 6 weeks after RCT. However, the repair groups with scaffold showed significant improvement compared with control groups. This study demonstrated the efficacy of an electroconductive fibrous scaffold in healing the RCT by improving the muscle atrophy in acute and sub-acute models [19, 126].

**Figure 5.**
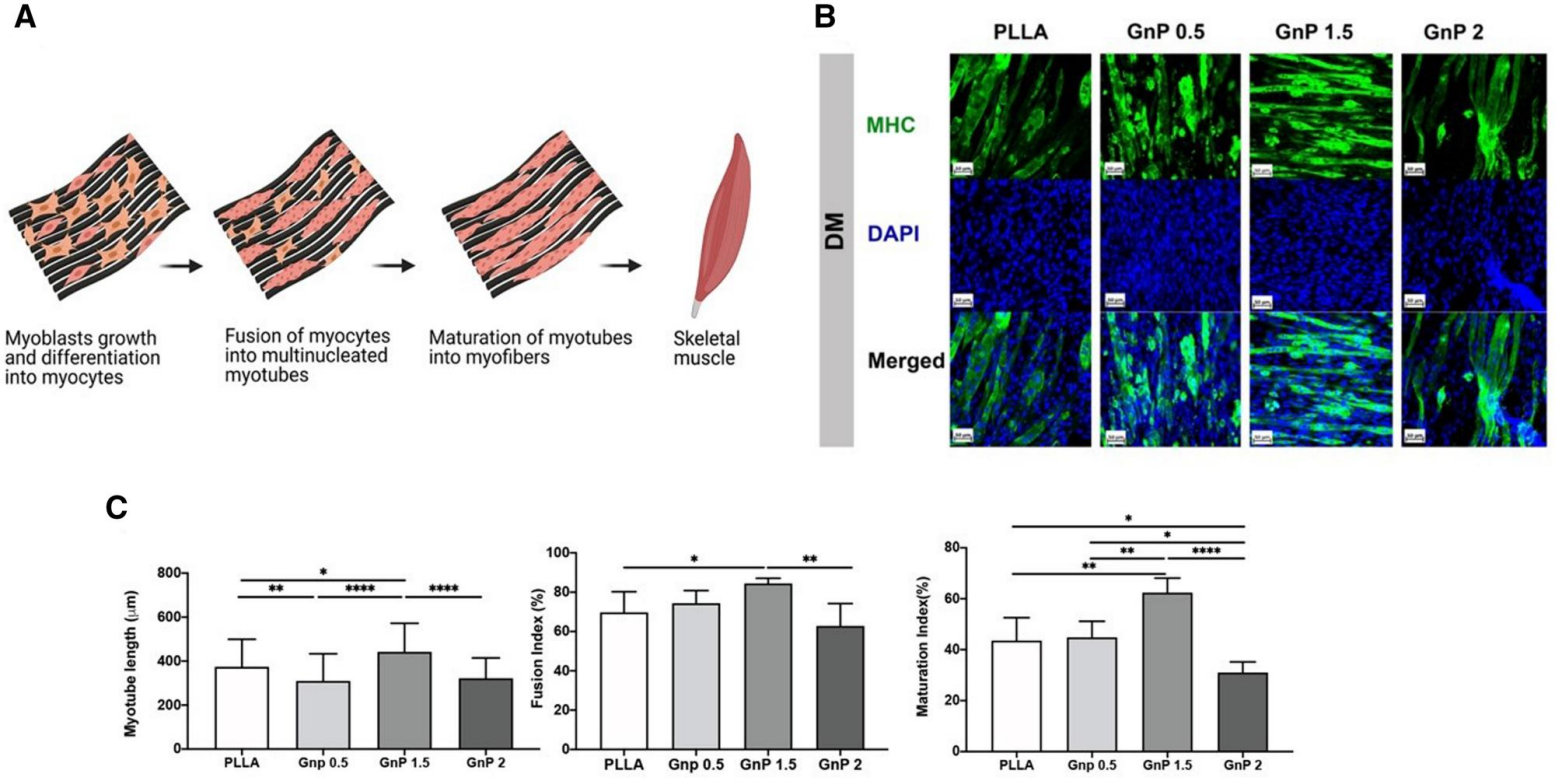
(**A**) Schematic illustration of muscle regeneration on graphene matrix, (**B**) immunofluorescent images of differentiated myotubes after 7 days on aligned matrices immune-stained for MHC and nucleus, (**C**) quantification of myotube length, fusion index, maturation index of differentiated myotubes after 7 days on aligned matrices [33].

Recently, our group reported the regenerative potential of graphene-based matrices to reverse muscle atrophy and reduce fat accumulation after chronic RCTs in a rat model [33]. We developed an EAM-based matrix by incorporating graphene nanoplatelets (GnPs) into aligned PLLA nanofibers. The GnP matrix significantly increased myotube formation of C2C12 myoblasts (Figure 5), which can be attributed to enhanced intracellular calcium ions in myoblasts as reported in the study [33]. The results also showed the efficiency of GnP matrices on the inhibition of fat formation *in vitro*.

**Figure 6.**
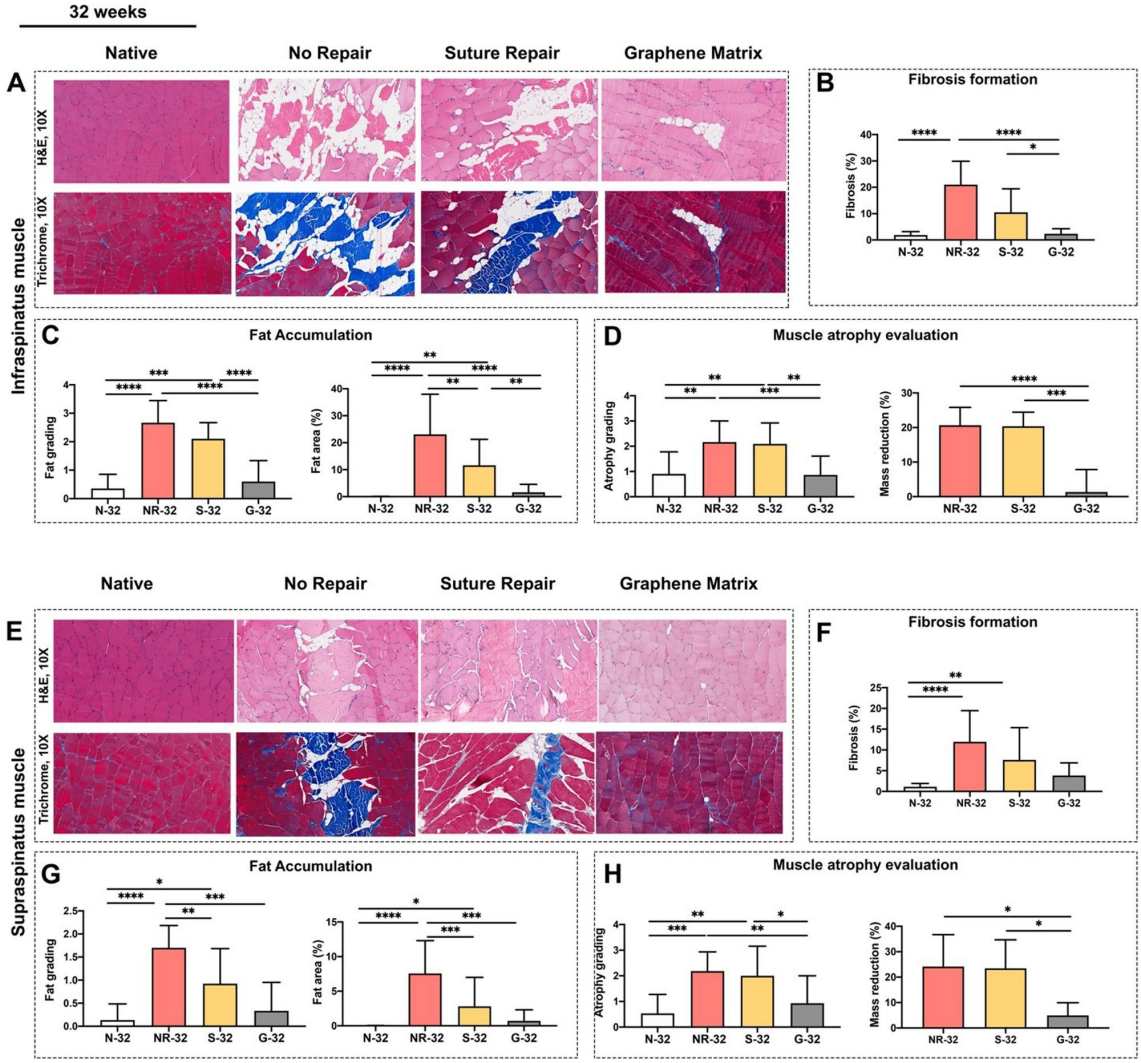
(**A**) The histological images of infraspinatus muscle at 32 weeks. The evaluation of (**B**) fibrosis formation, (**C**) fat accumulation, and (**D**) muscle atrophy at 32 weeks. (**E**) The histological images of supraspinatus muscle at 32 weeks. The evaluation of (**F**) fibrosis formation, (**G**) fat accumulation, and (**H**) muscle atrophy at 32 weeks. *P ≤ 0.05, **P ≤ 0.01, ***P ≤ 0.001, ****P ≤ 0.0001 [33].

Following the promising outcomes of the *in vitro* study, the matrices were implanted on the supraspinatus and infraspinatus muscles of chronic massive full-thickness RCTs in a rat model. The implantation of the matrices on the degenerated muscles demonstrated the ability of EAMs to reverse muscle degenerative changes *in vivo*. The histological evaluation exhibited the effects of the graphene-based matrix to reverse muscle atrophy, fat accumulation, and fibrosis in both supraspinatus and infraspinatus muscles at 24 and 32 weeks after the chronic massive full-thickness RCTs of the rat shoulder (Figure 6). The long-term biocompatibility of the matrix was also confirmed by pathological evaluation of internal organs. Furthermore, the study showed that reversing muscle degenerative changes improved the morphology and tensile properties of the tendon compared with current surgical techniques [33].

The ability of the graphene-based matrix to reverse muscle degeneration and the long-term biocompatibility of the matrices in an animal model provide convincing evidence for using the EAMs in future studies.

## Altering biologic factors

Several studies investigated different strategies to reduce muscle degeneration following the RCTs. Sevivas *et al.* reported the efficacy of the secretome of MSCs to prevent muscle degeneration in a rat chronic RCT model [236]. It was shown that the either systemic or local injection of MSCs secretome can reduce fat formation in a rat model of massive RCT [236]. Moreover, Wang *et al.* [237] demonstrated the preventive effects of local injection of adipose stem cell-derived exosomes on fat accumulation. The repair procedure was conducted in a rabbit RCT 6 weeks after the injury. The histological and biomechanical results showed a higher score of regeneration and strength compared with the control groups [237].

The efficacy of brown adipose tissue to promote muscle regeneration has been shown by Meyer *et al.* [238]. The results showed that transplantation of brown adipose tissue between the trapezius and supraspinatus muscles in a mouse model in conjunction with cardiotoxin injection to the supraspinatus muscle, significantly increased fiber cross-sectional area, muscle mass, and muscle function compared with the sham group [238]. These results showed that brown/beige adipocytes can be considered as a promising cell source for healing RCT. As mentioned earlier, many studies reported that FAPs are the main source of fat accumulation and atrophy after RCT. Gorski *et al.* [239], demonstrated the ability of FAPs to differentiate into UCP1-expressing brite adipocytes. The efficacy of local transplantation of these brite/beige adipocytes to reduce RC muscle degeneration in a mouse model has been demonstrated by Lee *et al.* [67]. The supraspinatus tendon and suprascapular nerve of mice were transected and repaired 2 or 6 weeks after the injury [67]. The repair procedures included: no treatment, PBS injection, or beige adipose FAPs injection. The samples were harvested 6 weeks after the repair surgery [67]. Consistent with other studies they reported fibrosis and fat accumulation after suture repair, confirming that current surgical repair cannot reverse muscle degeneration [67]. However, their results showed a significant reduction in fibrosis and fat accumulation in the beige adipose FAPs injection group [67]. The exact underlying mechanism is not clear however these promising results can be attributed to cytokine or other secreted factors by beige adipose FAPs, or the death and trans-differentiation of fibroblasts and adipocytes [67]. The group also reported the regenerative potential of beige FAPs in a massive RCT model [102]. The mice underwent supraspinatus, infraspinatus, and suprascapular nerve transection [102]. The repair procedure was performed 2 weeks after the first surgery. The treatment groups were no treatment, PBS injection, and beige FAPs injection into the supraspinatus muscles. The results showed a statistically significant reduction in fibrosis, muscle atrophy, and fat accumulation in the cell-treated group compared with control groups 6 weeks after the repair surgery [102].

**Figure 7.**
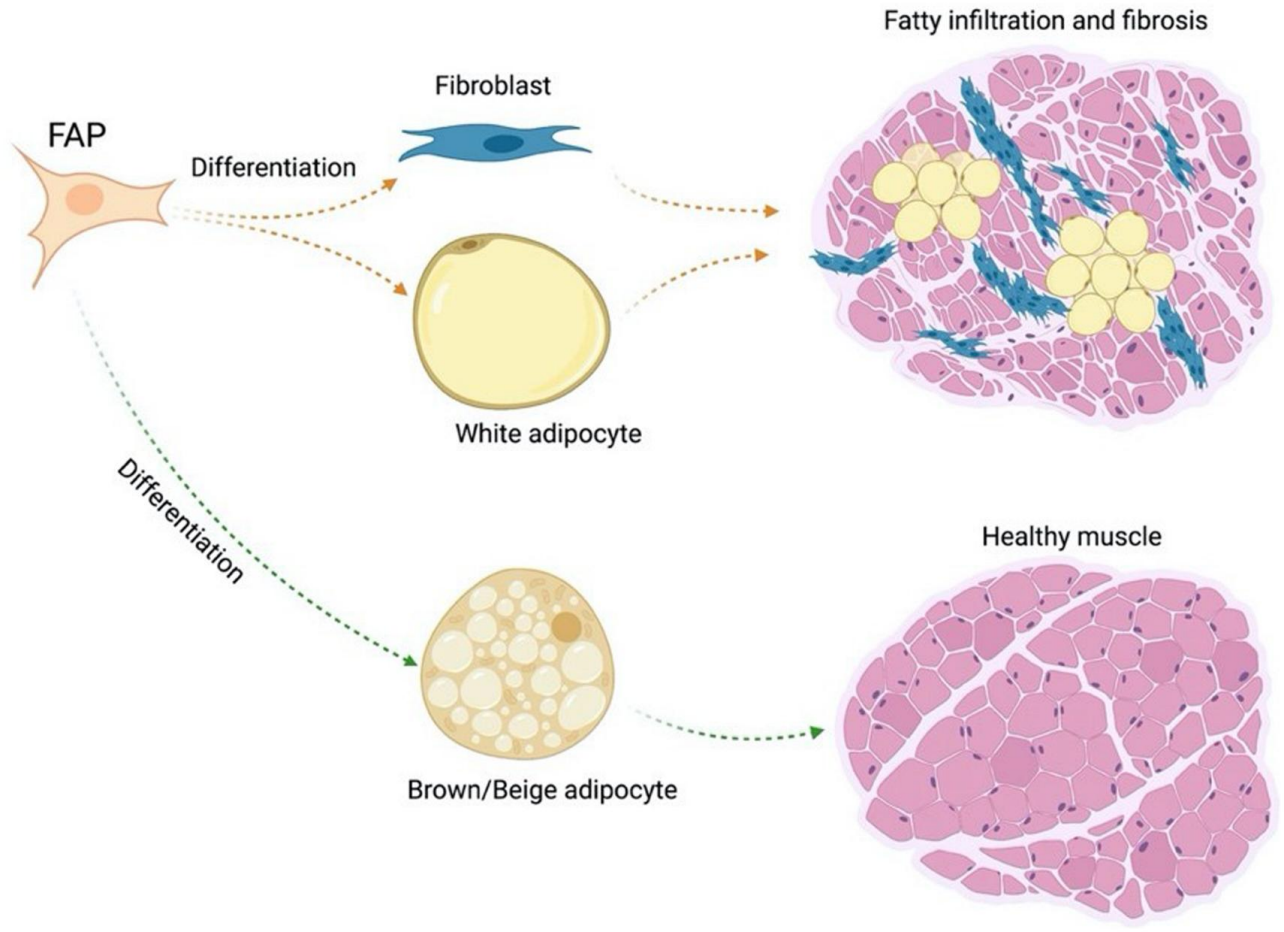
The schematic illustration of FAPs behavior. The underlying mechanism of fat accumulation and fibrosis formation is attributed to FAPs. However, the ability of FAPs to differentiate into brown/beige adipocytes can make it a novel therapeutic approach to reverse muscle degenerative changes after RCTs.

Taken together, the fat stores in torn RC can become a novel therapeutic approach to improve muscle conditions after RCT, as shown in Figure 7 [95]. Kong *et al.* reported the potential of brown adipose tissue to control skeletal muscle function by the secretion of significant quantities of myostatin into the blood [97]. The EAMs-based scaffolds can act as the vehicle to deliver the beige/brown adipocyte to the degenerated RC muscles. The studies showed the ability of EAMs to regenerate skeletal muscle and reverse RC fat accumulation and muscle atrophy. The combination of EAMs with beige/brown adipocytes can boost the RCT treatment and speed up the healing process.

**Figure 8.**
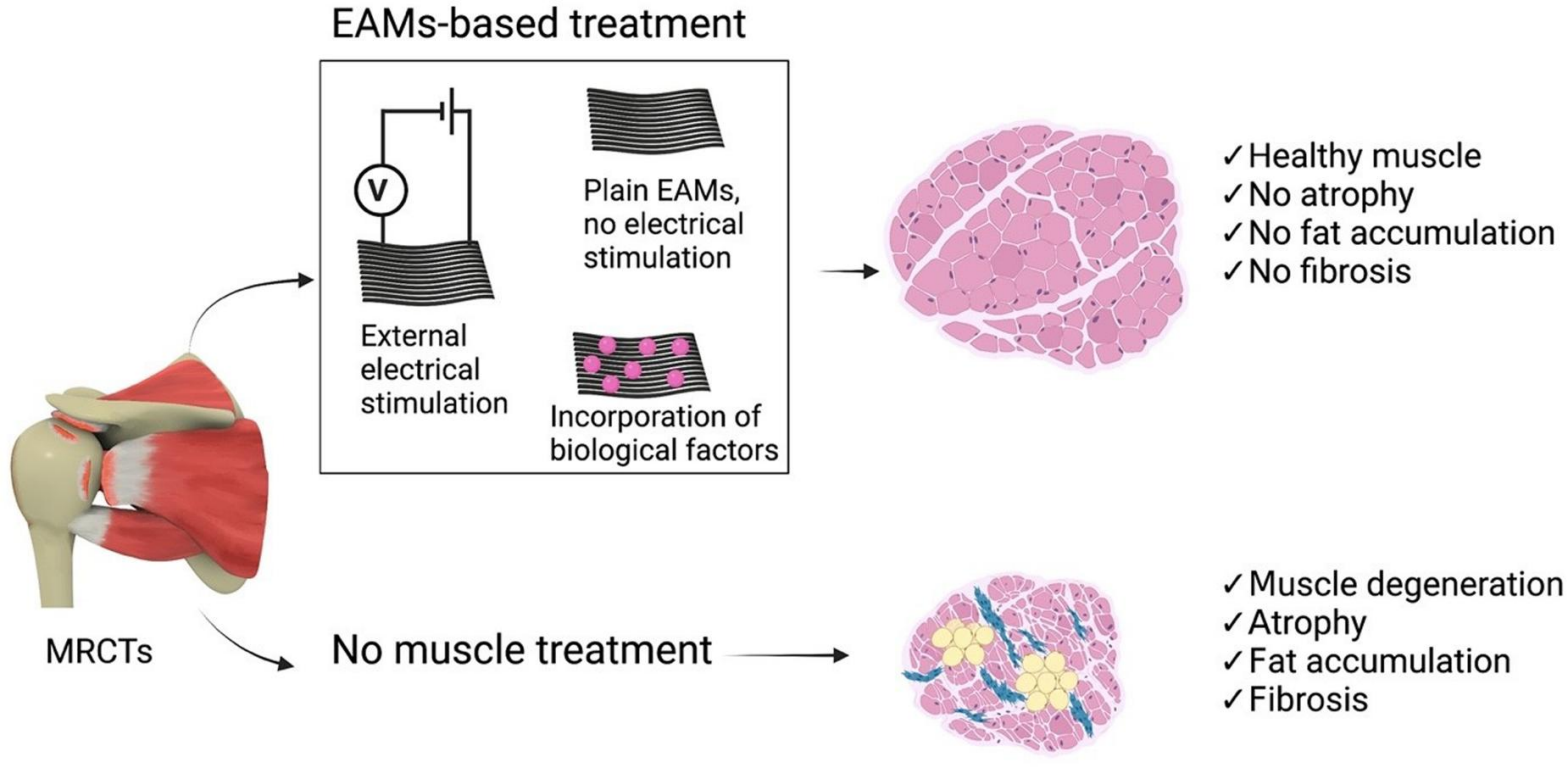
The schematic illustration showing the efficacy of EAMs either alone or in combination with ES and/or biological factors to reverse the muscle degeneration following RCTs.

Moreover, EAMs-based scaffold can be further developed by adding biological factors or small molecules such as signaling inhibitors. It has been shown that FGF-8b can induce myogenesis and inhibit adipogenesis [30]. Another study showed applying small molecule inhibitor of TGF-β signaling reduced RC muscle fibrosis and fatty infiltration in a mice RCT model [240]. Applying those molecules into EAMs can be a robust strategy to reverse the muscle degenerative changes following the chronic RCTs. The combination of an effective biological factor with EAMs can accelerate the treatment and have superior effects on the tendon-bone insertion regeneration and tensile properties (Figure 8).

## Limitations and challenges

Implementing electroconductivity in regenerative engineering and more precisely in RCT regeneration has its own challenges and limitations. External ES is beneficial for muscle tissue activity hence promoting healing, however, delivering this to the cells is a challenge. When using scaffolds in tissue engineering, cells will migrate to the porous structure as it will support and promote their proliferation and function, but if this substrate is not electrically conductive, the external stimuli will not affect where it is most needed, cells within the scaffold. Thus, there is a need for using electroconductive materials to facilitate this connection and pass on the external or the internal ESs. There have been a wide variety of electroconductive materials used in industry, however, one most important shortcoming of these is nonbiodegradability and poor processability. As a result, most of these EAMs are used alongside other biomaterials as coating, functionalized on the surface, or composites. This gives researchers an opportunity for tuning, altering, and manipulating the fabricated material to gain the desired properties such as biodegradability along electroconductivity. However, there is still a gap that needs to be filled in this regard and more studies focused on the mechanism of action, incorporation the best material in an optimized manner is needed. Moreover, heterogenous *in vivo* and clinical studies are essential for this field to unveil the long-term effect and mechanism of clearance of these EAMs in the body. Due to these limitations, and unknown reactions to electrically conductive materials in the body, this studies have not been able to reach to clinical studies on RC injuries.

## Conclusions

In this review, we discussed the biological mechanisms of RC muscle degeneration, then reviewed the applications of EAMs for skeletal muscle regeneration including RCT treatment. Various tissues are affected as a result of RCT injury which leads to a high retear rate after surgery. The current clinical strategies have failed to revert muscle degeneration and regenerative engineering is a promising approach to address the challenges. The endogenous electrical potential has a key role during the wound-healing process. By adding external ES, it has been shown to enhance the cellular activity of various types of cells such as MSCs, fibroblasts, and myoblasts. Therefore, researchers have developed and investigated EAMs to enhance local ES for the regeneration of electrically excitable tissue types such as skeletal muscle. Both *in vitro* and *in vivo* studies suggest the strong potential for the use of EAMs for the regeneration of skeletal muscles, although there is still room for improvement, including scaffold stiffness and conductivity without suppressing the potential for muscle regeneration. There are few studies that have examined the efficacy of EAMs for RCT treatment. However, our recent study demonstrated that EAMs application reverted muscle degeneration, not only muscle atrophy but also fibrosis formation and fat accumulation. Thus, it is beneficial to fabricate a scaffold or an augmentation that can be electrically stimulated. Then by implanting it in the injured RC, especially in contact with the muscle, it can act as a signaling facilitator and help repair the injury by improving muscle contractibility, reduce fat accumulation and fibrosis and improve regeneration in the area of effect. In addition, the use of biological factors such as secretome, brown/beige adipocytes, and growth factors showed promising results for the RCT treatment. EAMs can serve as a desirable carrier of those biological factors. In future studies, it is critical to focus on the efficient and controlled delivery by providing optimized electrical field to boost the regeneration of complex RC tissues, which will be a novel clinical strategy for the treatment of RCT.
